# Draft Genome Sequence of *Trueperella pyogenes*, Isolated from the Infected Uterus of a Postpartum Cow with Metritis

**DOI:** 10.1128/genomeA.00194-14

**Published:** 2014-04-24

**Authors:** Robert J. Goldstone, Matt Amos, Richard Talbot, Hans-Joachim Schuberth, Olivier Sandra, I. Martin Sheldon, David G. E. Smith

**Affiliations:** a Institute of Infection, Immunity and Inflammation, College of Medical, Veterinary and Life Sciences, University of Glasgow, Glasgow, United Kingdom; b Institute of Life Science, School of Medicine, Swansea University, Singleton Park, Swansea, United Kingdom; c Roslin Institute, University of Edinburgh, Easter Bush, Midlothian, Scotland, United Kingdom; d Immunology Unit, University of Veterinary Medicine, Hannover, Germany; e INRA, UMR1198 Biologie du Développement et Reproduction, Jouy-en-Josas, France; f ENVA, Maisons Alfort, France; g Moredun Research Institute, Pentlands Science Park, Bush Loan, Edinburgh, Midlothian, United Kingdom

## Abstract

*Trueperella pyogenes* is a common commensal bacterium and an opportunistic pathogen associated with chronic purulent disease, particularly in ruminants. We report here the genome sequence of a *T. pyogenes* isolate from a severe case of bovine metritis. This is the first full record of a *T. pyogenes* genome.

## GENOME ANNOUNCEMENT

Recently, the genus *Trueperella* was proposed to encompass 5 species previously classified as belonging to *Arcanobacterium* (1). Among these reclassified species is *T. pyogenes*, previously known as *Arcanobacterium pyogenes, Actinomyces pyogenes*, and *Corynebacterium pyogenes*. *T. pyogenes* has been long recognized as a mucosal membrane resident in many animal species and as an opportunistic pathogen (2). *T. pyogenes* is particularly associated with suppurative infections, such as mastitis, septic arthritis, liver abscessation, pneumonia, endometritis, and metritis. *T. pyogenes* is recognized as a key etiological agent in bovine endometritis and metritis, frequently in conjunction with other bacteria, including *Escherichia coli*, *Fusobacterium necrophorum*, *Fusobacterium nucleatum*, and *Prevotella* spp. (3), although *T. pyogenes* correlates with most severe disease presentation and pathology (4). Despite the bacterium's being known as an opportunistic pathogen for many decades, the understanding of *T. pyogenes* remains rudimentary. No genome sequences for *T. pyogenes* had been available in the public domain; therefore, we carried out the sequencing of this genome.

*T. pyogenes* MS249 was isolated from the uterus of an animal with a severe case of metritis in which disease persisted and progressed to endometritis (5). Genomic DNA was prepared using the DNeasy blood and tissue kit (Qiagen), according to the manufacturer’s instructions. Sequencing was carried out using 454 GS-FLX and Illumina GA IIx 130-bp paired-end sequencing. The reads were assembled using Velvet (6) and the Celera Assembler with the Best Overlap Graph (CABOG) (7), and gaps were closed using unmapped 454 and Illumina reads. The prediction of open reading frames (ORFs) was achieved using FgenesB (8) via the SoftBerry web interface (http://linux1.softberry.com/berry.phtml), rRNA prediction was carried out via the HMMER Web server (9), and tRNA prediction was done using tRNAscan-SE via the WebMGA interface (10). The assembled 248 contigs totaled 2,236,677 bp, with a G+C content of 59.8%. The genome was predicted to contain 4 rRNA operons, 47 tRNAs, and 2,095 protein-coding sequences (CDSs).

The main recognized virulence factor of *T. pyogenes* is pyolysin (PLO), an exported, cholesterol-dependent, pore-forming cytotoxin (CDC) (11–13), and the PLO gene sequence was identical to that published for other strains of *T. pyogenes*. Additional virulence-associated factors in MS249 included fimbriae/pili (Fim/Pil), collagen-binding protein (CbpA) (14), and neuraminidases (NanH and NanP) (2). Multiple *fim* genes have been reported, the presences of which are variable among strains (14, 15), and in strain MS249, *fimA* and *fimC* were identified, along with an additional *fimG* locus.

Resistance to antibiotics often used in veterinary practice is common in *T. pyogenes*. A gene encoding resistance to tetracycline (*tetW*) was identified in the genome of strain MS249. Although resistance to macrolides, chloramphenicol, and β-lactam antibiotics among *T. pyogenes* strains has been reported (16–20), no specific resistance elements were identified in strain MS249, consistent with its sensitivity to these antibiotics.

This is the first reported genome sequence for *T. pyogenes*. This sequence will be used to extend the understanding of this novel bacterial opportunistic pathogen and form the basis for systematic studies of its pathogenicity and physiology, as well as to perform a comparative genomic analysis among *Actinobacteria*.

### Nucleotide sequence accession numbers.

This whole-genome shotgun project has been deposited at DDBJ/EMBL/GenBank under the accession no. JALQ00000000. The version described in this paper is version JALQ01000000.
